# Antibiotic prophylaxis in oral implant surgery in Germany: a cross-sectional study

**DOI:** 10.1186/s40729-024-00577-4

**Published:** 2024-12-16

**Authors:** Jens-Uwe Peter, Johannes Ladewig, Christian Stoll, Oliver Zolk

**Affiliations:** 1 Institute of Clinical Pharmacology, Immanuel Hospital Rüdersdorf, Brandenburg Medical School Theodor Fontane, Rüdersdorf, Germany; 2 Department of Oral, Craniomaxillofacial and Plastic Surgery, Faculty of Medicine, University Hospital Ruppin-Brandenburg, Brandenburg Medical School Theodor Fontane, Neuruppin, Germany

**Keywords:** Anti-bacterial agents, Antibiotic prophylaxis, Dental implants, Germany, Drug prescriptions, Surveys and questionnaires, Dental health services

## Abstract

**Purpose:**

Prophylactic antibiotics are used in dental implants to reduce infection risk and implant failure, especially benefiting patients with risk factors. However, evidence suggests that using clindamycin or extending antibiotics postoperatively has an unfavorable risk–benefit ratio.

**Methods:**

This national cross-sectional study analyzed antibiotic prophylaxis during implant insertion across Germany. Dentists from the German Society for Oral Implantology (DGOI) provided demographic information and data on the next 10 consecutive implant patients, including age, sex, risk factors, type of implantation, and antibiotic details.

**Results:**

103 dentists participated, providing data on 1040 patients. Most dentists were male and aged 30–64. Patients were evenly split between genders, with an average age of 51 years. Antibiotics were administered in 87.6% of all cases, more frequently for patients undergoing bone augmentation (OR 7.01, p < 0.0001), immediate (OR 3.11, p = 0.002) or delayed (OR 5.30, p < 0.0001) implant insertion, and those with cardiovascular disease (OR 3.24, p = 0.009). 74.8% of implantologists tended to use antibiotic prophylaxis routinely, while the remaining implantologists decided on a case-by-case basis. Implantologists primarily used aminopenicillins for 63.8% of prescriptions and clindamycin for the remaining 35.6%. Additionally, 78.8% of patients with prophylaxis received postoperative, multi-day treatments.

**Conclusions:**

The study reveals extensive antibiotic use for perioperative prophylaxis in implant surgery, often not justified by current recommendations, particularly concerning the choice of antibiotic (e.g., clindamycin) and duration (e.g., postoperative use). Specialized clinical guidelines and targeted training for dentists on antibiotic prophylaxis are needed.

**Graphical Abstract:**

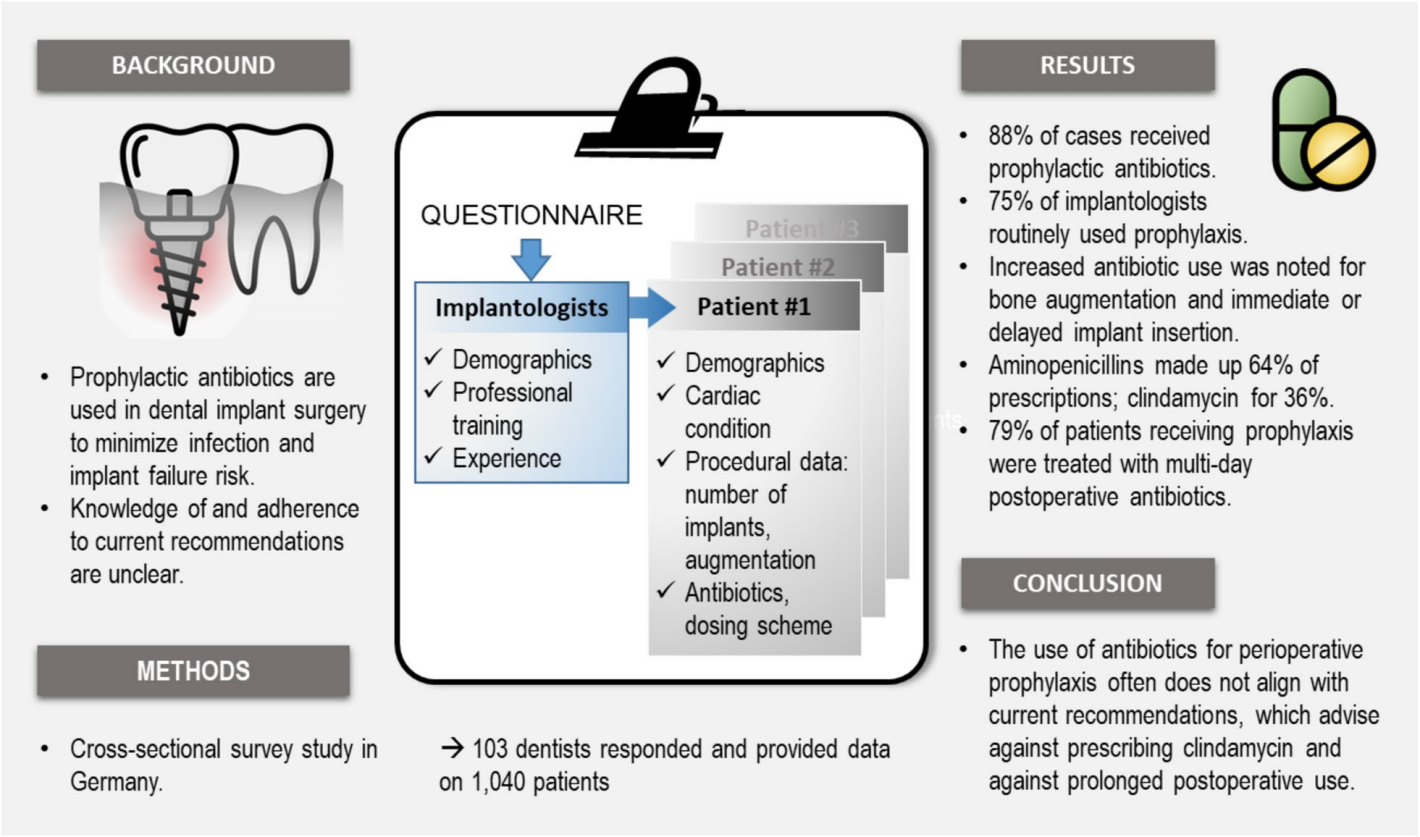

## Background

Advances in implant research and technology have transformed the replacement of missing teeth with endosseous implants into the standard of care, making implant-supported prostheses the primary and enduring solution for oral rehabilitation [1]. Nowadays, dental implants are frequently utilized to fulfill both patient and practitioner preferences for aesthetics, chewing function, and long-term durability [2]. However, a small percentage of dental implants, approximately 7%, may fail, classified as either “early” or “late” depending on whether they occur before or after occlusal loading with a prosthetic superstructure, respectively [3]. In a study with a long follow-up period for all implants, the 7-year and 10-year survival rates—indicating whether the implant was still in place—were 95.87% and 92.6%, respectively [4]. Despite the low rate of implant loss, a significant number of late complications were noted, with peri-implant mucositis occurring in 39.7% of patients and peri-implantitis in 16.7% [4]. Early implant failures, most common, are often attributed to issues with osseointegration due to local and/or systemic factors. Potential risk factors for early failure may include bone quality and volume, site characteristics, and a greater number of implants placed per patient, as well as systemic factors such as smoking and diabetes mellitus [5–7]. Infections have also been identified as critical factors influencing osseointegration. Postoperative infections, though rare, can occur within the first month after implant placement, with reported prevalence reaching approximately 10% [8]. In a retrospective cohort study involving 474 outpatients with a total of 1625 implants, postoperative infections were observed in 6.5% of the patients and 1.7% of the implants [9]. Treating such complications can be complex, and infection may persist until the implant is removed. Since the early days of oral implantology, preventive antibiotics have been integrated into implant placement protocols to control the presence of various oral pathogenic bacterial species that may contribute to postoperative infections and consequently implant failure. Limited evidence from randomized controlled trials, each with a small patient cohort, and their meta-analyses indicates that the number needed to treat (NNT) with antibiotic prophylaxis to prevent implant failure ranges from 14 to 55 [10–13]. Additionally, to prevent one patient from developing postoperative infection, the NNT is 143 [11].

Patient populations vary, and the effectiveness of antibiotic prophylaxis depends on the individual patient’s preprocedural risk. It may be more effective in “complex” cases, such as those with compromised immune systems, grafting procedures, or immediate placement in extraction sockets, compared to “straightforward” cases [14]. Despite its potential benefits for high-risk patients, prophylactic antibiotics in dental implantology can cause adverse reactions, including life-threatening allergies, toxic effects on organs, and harm to gut microflora. The number needed to treat to prevent one adverse side effect is about 528 [13]. Additionally, overuse and misuse of antibiotics can promote bacterial resistance. Consequently, the European Association for Osseointegration does not recommend antibiotic prophylaxis for “straightforward” implant surgery in healthy patients [14]. However, the Spanish Society of Implants recommends it for all implant surgical procedures except the prosthetic phase [15].

There is less consensus on antibiotic prophylaxis in implant surgery compared, for example, to endocarditis prophylaxis. Clear guidelines and protocols for dental implantology are largely lacking. The only current German guideline recommends a single preoperative antibiotic dose for diabetic patients during implant surgery but does not specify which antibiotic to use [16]. The 2015 4th European Association for Osseointegration (EAO) Consensus Conference advised against antibiotic prophylaxis for low-risk procedures in healthy patients, suggesting it is only beneficial for complex cases or compromised patients [14], In contrast, the Spanish Society of Implants (SEI) recommends antibiotic prophylaxis even for routine procedures in healthy patients [17].

Recent studies have investigated antibiotic prescribing habits for oral implant surgery in various countries [18, 19], but comparative data from Germany are missing. To address this gap, we conducted a survey in Germany. Unlike previous surveys, we asked implantology colleagues to prospectively report on the treatment of 10 consecutive patients who underwent implant surgery in their practice. This approach provided a more realistic and unfiltered picture of antibiotic prophylaxis use in implant surgery.

## Methods

### Study design

The study was approved by the Ethics Committee of Brandenburg Medical School Theodor Fontane (file number E-01-20220107). The study involves a prospective questionnaire survey, with all participants informed about the use of their data for research purposes. It adheres to the Strengthening the Reporting of Observational Studies in Epidemiology (STROBE) guidelines for prospective cohort studies and follows the World Medical Association’s Declaration of Helsinki. All data were anonymized, ensuring that the identity of respondents or patients could not be determined at any point during data collection and analysis.

### Study population and procedure

The sample comprised dentists registered as implantologists with the German Society of Oral Implantology (DGOI). A questionnaire was sent to their registered addresses or completed by personal interview. Inclusion criteria required active involvement in implantology and DGOI registration, with no defined exclusion criteria.

The questionnaire had two sections. The first section collected anonymized demographic data of the participating dentists, including gender, age, federal state of practice, oral surgery training status, and the number of implant surgeries they perform annually. The second section gathered information on 10 consecutive implant patients, regardless of antibiotic prophylaxis use. For each patient, the following data were collected:

1. Patient demographics: age (in years) and sex.
2. Cardiovascular conditions: presence of pre-existing conditions.
3. Antibiotic use:
  Whether perioperative antibiotics were prescribed.
  If prescribed:
    Indication for use:
      Implant insertion.
      Endocarditis prophylaxis.
      Both implant insertion and endocarditis prophylaxis.
    Name and dosage of the antibiotic.
    Timing of administration (preoperatively, postoperatively, or both).
    Dosing schedule and duration of therapy.
4. Procedural information:
  Number of implants placed.
  Whether bone augmentation was performed, and type of augmentation:
    Sinus floor elevation (sinus lift).
    Horizontal augmentation (attachment osteoplasty).
    Vertical augmentation (support osteoplasty).
  Timing of implant insertion relative to tooth extraction:
    Immediate (same session as tooth extraction).
    Early (up to 6 weeks after tooth extraction).
    Late (more than 6 weeks after tooth extraction).

### Statistics

All statistical analyses were performed using IBM SPSS Statistics version 27. Continuous variables were summarized using means and standard deviations, as well as medians and interquartile ranges; categorical variables were summarized using frequencies and percentages. Patient characteristics were compared using the Pearson Chi-square test for categorical variables. For comparison of continuous variables between two groups, the independent samples t-test was used for parametric tests, given that the variables followed an approximately normal distribution. Otherwise, the non-parametric Mann–Whitney U test was employed. For the analysis of one binary outcome variable (i.e., whether antibiotic prophylaxis was used) and multiple independent variables, multivariable regression models were calculated. Independent variables significantly associated with the use of antibiotic prophylaxis in univariable comparisons were included in the regression model. Multinomial logistic regression analysis was used to investigate the dependence of the choice of a specific antibiotic class (i.e., aminopenicillin, aminopenicillin with beta-lactamase inhibitor, or clindamycin) for perioperative prophylaxis on the independent variables. Results of regression analyses are displayed as odds ratios (OR) with confidence intervals (CI). The significance level was set at p < 0.05.

## Results

**Fig. 1:**
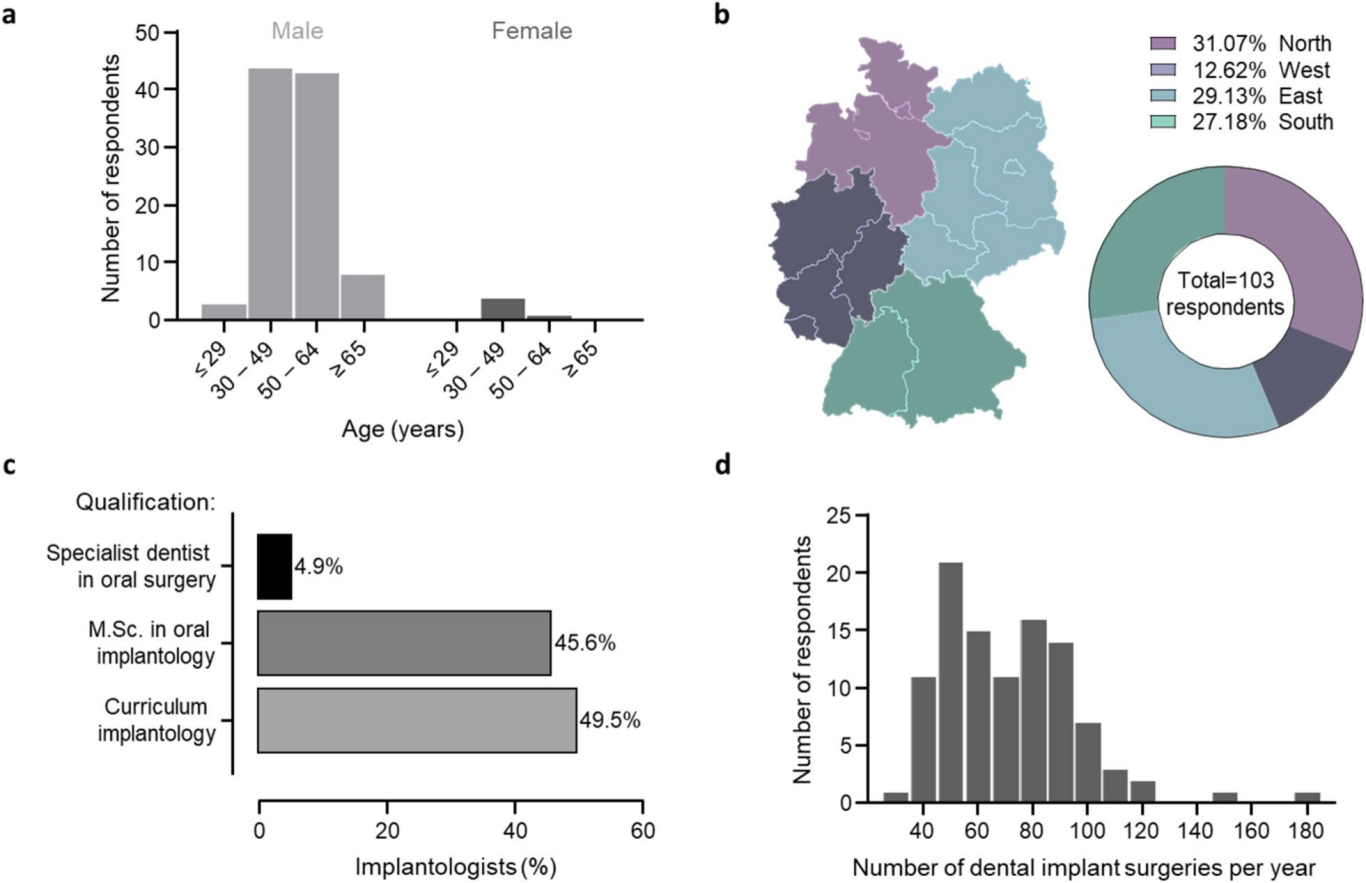
Implantologists’ characteristics: age and sex (**a**), region (**b**), qualification (**c**), and number of implant surgeries per year (**d**)

Data were collected from March 2022 to October 2022. A total of 279 dentists working in implantology were contacted; 176 did not respond, and 103 returned completed questionnaires, resulting in a 37% response rate. Data from these 103 practitioners were analyzed. Most implantologists were aged 30–49 (n = 48; 46.6%) and male (n = 98; 95.1%) (Fig. 1a). Western Germany was slightly underrepresented, while the other three regions of Germany had a more even distribution of respondents (Fig. 1b). Six respondents had oral surgery training (specialist dentist in oral surgery); most had completed either the implantology curriculum or obtained a Master of Science degree in oral implantology in roughly equal proportions (Fig. 1c). Implantologists reported performing an average of 69.9 ± 24.9 implantation surgeries annually (median 68), with a range of 30 to 180 surgeries per year (Fig. 1d).

**Fig. 2:**
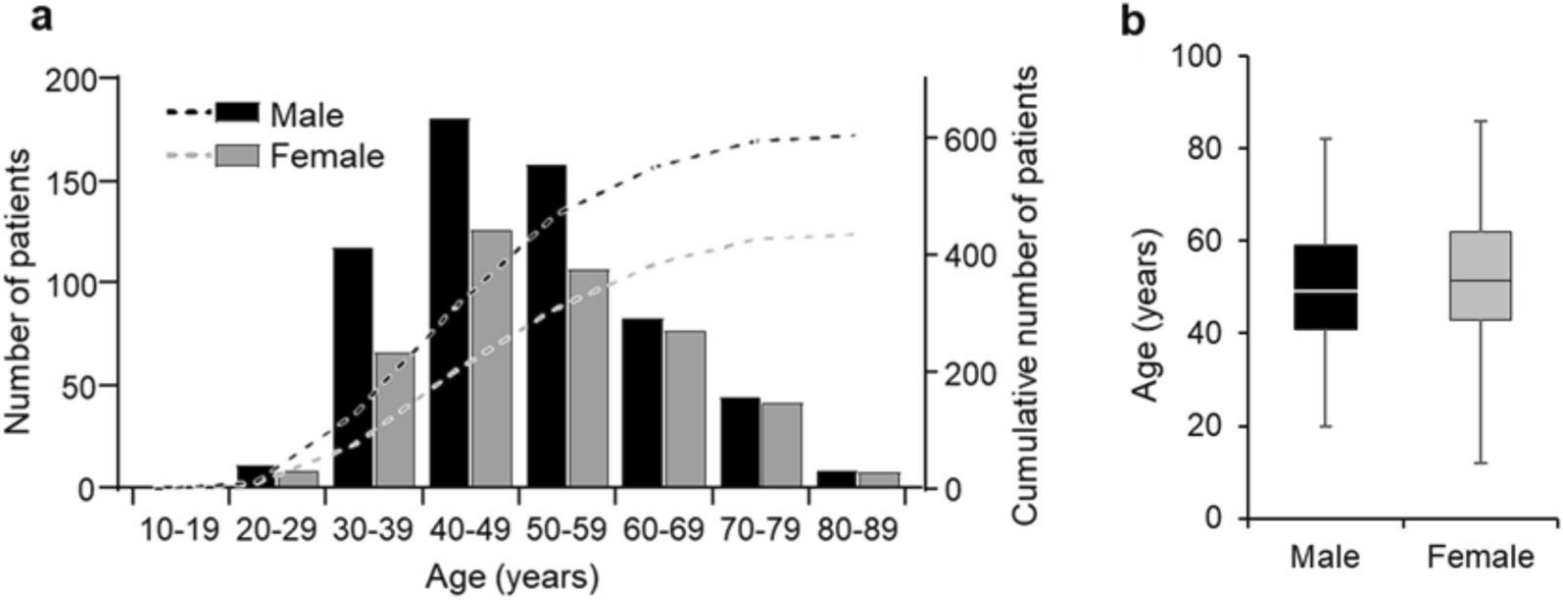
Patients’ characteristics: number of patients by age group and sex (**a**), age distribution of patients by sex (**b**) boxplot shows median, IQR, minimum, and maximum age of both sexes

**Fig. 3:**
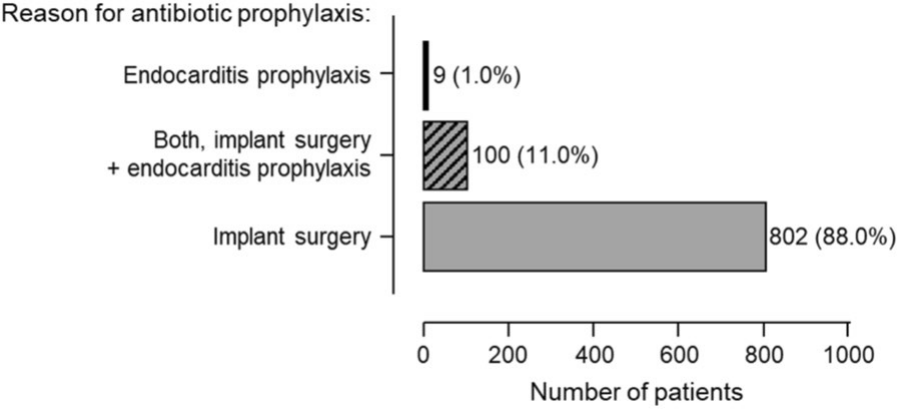
Implantologists’ rationale for antibiotic prophylaxis

**Fig. 4:**
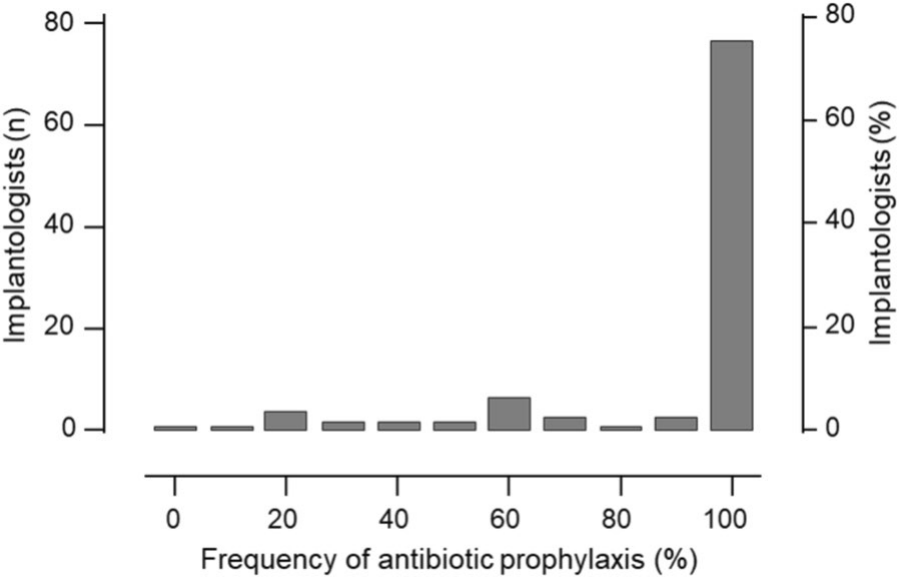
Histogram of antibiotic prophylaxis use among implantologists, based on samples of at least 10 patients each

Implantologists were asked to anonymously provide medical treatment data for 10 consecutive implant patients. Except for one implantologist who provided data for 20 patients, each provided data for 10 patients, totaling 1040 records. The mean age of patients was 51.3 ± 12.7 years (range 12–86 years), with more males (58.1%) than females (41%) (Fig. 2). Antibiotic prophylaxis was provided to 911 patients (87.6%). As shown in Fig. 3, in most cases (88%), it was administered solely due to implant surgery, in 1% solely for endocarditis prophylaxis, and in 11% for both implant surgery and endocarditis prophylaxis. 74.8% of the implantologists surveyed administered antibiotic prophylaxis to all their patients (Fig. 4). Among the others, the prescription frequency ranged from 0 to 90%. Univariable analysis revealed that preexisting cardiovascular disease, the number of implants inserted, whether bone augmentation was performed, and the time after tooth extraction significantly influenced the decision on antibiotic prophylaxis (Table 1). The analysis also revealed that the implantologist’s qualification significantly affected the likelihood of prescribing antibiotic prophylaxis (Table 2).

**Table 1:** Univariable analysis of patient and procedural factors influencing the decision on antibiotic prophylaxis

	Antibiotic prophylaxis		P value
	Yes (n = 911)	No (n = 129)	
Age (years)			
[mean ± SD]	51.45 ± 12.59	49.81 ± 13.29	0.066^#^
[median (IQR)]	50.0 (43.0–61.0)	47.0 (41.0–87.5)	
Sex [n (%)]			
Female	525 (86.9%)	79 (13.1%)	0.437*
Male	386 (88.5%)	50 (11.5%)	
Cardiovascular disease [n (%)]			
No	791 (86.5%)	123 (13.5%)	0.006*
Yes	120 (95.2%)	6 (4.8%)	
Number of implants			
[mean ± SD]	1.82 ± 1.19	1.52 ± 0.88	0.003^#^
[median (IQR)]	1.0 (1.0–2.0)	1.0 (1.0–2.0)	
Number of implants [n (%)]			
1	474 (84.8%)	85 (15.2%)	0.003*
≥ 2	437 (90.9%)	44 (9.1%)	
Augmentation [n (%)]			
No	533 (82.0%)	117 (18.0%)	< 0.001*
Yes	378 (96.9%)	12 (3.1%)	
Horizontal	40 (93.0%)	3 (7.0%)	
Sinus lift	72 (97.3%)	2 (2.7%)	
Vertical	266 (97.4%)	7 (2.6%)	
Implantation time after tooth extraction [n (%)]			
Immediate	70 (79.5%)	18 (20.5%)	< 0.001*
Early	66 (68.0%)	31 (32.0%)	
Delayed	775 (90.6%)	80 (9.4%)	

*SD* standard deviation, *IQR* interquartile range

*Chi-squared test

^#^Mann–Whitney U test

**Table 2:** Univariable analysis of implantologist factors influencing the decision on antibiotic prophylaxis

	Antibiotic prophylaxis		P value
	Yes (n = 911)	No (n = 129)	
Implantologists’ age (years) [n (%)]			
≤ 29	30 (100%)	0 (0%)	0.078*
30–49	414 (86.3%)	66 (13.8%)	
50–64	400 (88.9%)	50 (11.1%)	
≥ 65	67 (83.8%)	13 (16.3%)	
Implantologists’ qualification [n (%)]			
Curriculum implantology	430 (84.3%)	80 (15.7%)	< 0.001*
M.Sc. in oral implantology	433 (92.1%)	37 (7.9%)	
Specialist dentist in oral surgery	48 (80.0%)	12 (20.0%)	
Number of implant surgeries performed by implantologists per year			
[mean ± SD]	70.1 ± 25.2	68.0 ± 21.2	0.654^#^
[median (IQR)]	67 (50–85)	70 (40–85)	

*SD* standard deviation, *IQR* interquartile range

*Chi-squared test

^#^Mann–Whitney U test

**Fig. 5:**
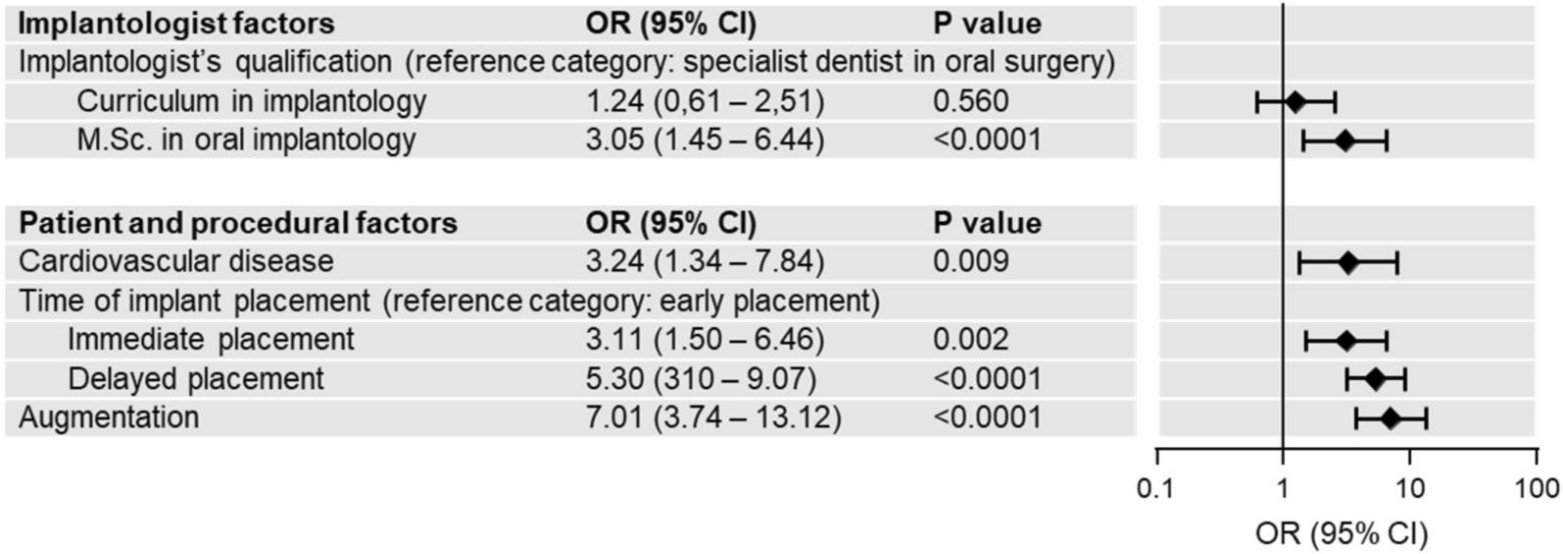
Binary logistic regression analysis of factors influencing the decision on antibiotic prophylaxis in implant surgery; *OR* odds ratio, *CI* confidence interval

The multivariable logistic regression model (Fig. 5) identified four factors significantly associated with the prescription of antibiotic prophylaxis: the implantologist’s qualification (OR 3.05, p < 0.0001 for M.Sc. in oral implantology vs. specialist dentist in oral surgery), the patient’s diagnosis of a cardiovascular disease (OR 3.24, p = 0.009), whether augmentation was performed (OR 7.01, p < 0.0001), and whether the dental implant insertion was immediate (OR 3.11, p = 0.002) or delayed (OR 5.30, p < 0.0001) compared to early insertion.

Nine patients with implants inserted by seven different implantologists received antibiotics solely for endocarditis prophylaxis. In three cases, a single dose of 1000 mg amoxicillin was used 60 min before surgery. In the remaining six cases, 600 mg clindamycin was used: a single dose 60 min before surgery in one case and repeated doses for 2–4 days, starting 60–120 min before surgery, in five cases.

Table 3 summarizes the drugs and dosage levels for the 902 cases that received antibiotics for the prophylaxis of dental implant infections, either solely for this purpose (802 cases) or in combination with endocarditis prophylaxis (100 cases). Approximately two-thirds of the patients received an aminopenicillin, with or without a beta-lactamase inhibitor, while another third received the lincosamide clindamycin. Only 5 patients received a different antibiotic, namely the tetracycline doxycycline.

**Table 3:** Antibiotic drugs used for prophylaxis of dental implant complications

Antibiotic drug class	n	(%)	Antibiotic drug and dose level	n	(%)
Aminopenicillins	481	(53.3%)	Amoxicillin 1000 mg	468	(51.9%)
			Amoxicillin 750 mg	9	(1.0%)
			Amoxicillin 500 mg	4	(0.4%)
Aminopenicillins + BLI	95	(10.5%)	Amoxicillin/clavulanic acid 875 mg/125 mg	93	(10.3%)
			Ampicillin/sulbactam 4000 mg/2000 mg	2	(0.2%)
Lincosamides	321	(35.6%)	Clindamycin 600 mg	310	(34.4%)
			Clindamycin 300 mg	11	(1.2%)
Tetracyclines	5	(0.6%)	Doxycycline 100 mg	5	(0.6%)

*BLI* beta-lactamase inhibitor

Multinomial logistic regression analysis revealed that, among all factors tested—such as the implantologist’s age and qualifications, the patient’s age and sex, augmentation, time of implant insertion (immediate, early or delayed) and number of implants—only the decision to perform bone augmentation significantly influenced the choice of antibiotic class (Table 4). Patients who underwent bone augmentation had 1.8 times higher odds of receiving an aminopenicillin with a beta-lactamase inhibitor and 0.5 times lower odds of receiving clindamycin compared to those receiving aminopenicillin without a beta-lactamase inhibitor.

**Table 4:** Multinomial logistic regression model of factors influencing the choice of antibiotic class

	Aminopenicillin (n = 480)	Aminopenicillin + BLI (n = 96)	Clindamycin (n = 321)	Doxycycline (n = 5)	p value (Chi-square)
Augmentation					
No	265 (50.2%)	39 (7.4%)	224 (42.4%)	0 (0.0%)	< 0.0001
Yes	215 (57.5%)	57 (15.2%)	97 (25.9%)	5 (1.3%)	
Multinomial regression analysis					
OR (95% CI)	Reference	1.8 (1.2–2.8)	0.5 (0.4–0.7)	n.d	
p-value		0.0096	< 0.0001		

*OR* odds ratio, *CI* confidence interval, *BLI* beta-lactamase inhibitor, *n.d.* not determined

**Fig. 6:**
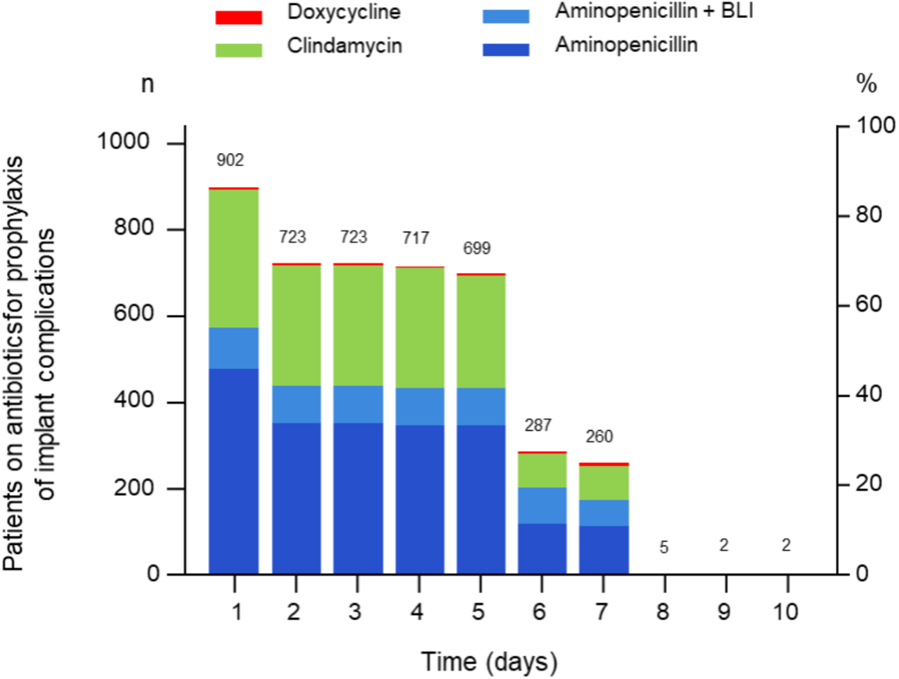
Duration of antibiotic prophylaxis for each antibiotic; *BLI* beta-lactamase inhibitor

Unlike the other data, the dosing scheme was not completely reported for all patients. The duration of antibiotic treatment for those who received antibiotics for prophylaxis of dental implant complications is summarized in Fig. 6 and Table 5. Most patients (77.5%) received antibiotic prophylaxis as a multi-day treatment for 5 or more days. In the multi-day treatment cases, the first dose was administered before surgery in 81.3% of patients, while 18.7% started antibiotics post-surgery. Only 18.8% received a single preoperative dose as prophylaxis.

**Table 5:** Frequency of antibiotic drugs used for prophylaxis of dental implant complications in different dosing regimens

Dosing regimen	Total n (%)	Amino-penicillin (n)	Aminopenicillin + BLI (n)	Clindamycin (n)	Doxycycline (n)
Single dose pre-OP	170 (18.8%)	123	9	38	0
Single dose post-OP	1 (0.1%)	1	0	0	0
Pre- and post-OP doses at day of surgery	6 (0.7%)	1	0	5	0
Multi-day treatment, start pre-surgery	578 (64.1%)	275	87	214	2
Multi-day treatment, start post-surgery	133 (14.7%)	68	0	62	3
Missing data	14 (1.6%)	10	0	4	0

*BLI* beta-lactamase inhibitor

## Discussion

Dental implant placement is a clean-contaminated (class II) surgical procedure that involves the risk of bacterial contamination during insertion and subsequent biofilm formation, which can lead to non-integration and implant loss [20, 21]. Infected biomaterials like titanium or zirconia are often resistant to antibiotics that are normally effective against periodontal bacteria, often necessitating implant removal [20]. Infection risk can be influenced by the surgeon’s skill and maintenance of asepsis during surgery. Additionally, perioperative antibiotic prophylaxis can help reduce the risk of periimplantitis and implant loss. The effectiveness of prophylactic antibiotics in implant surgery has been studied in various randomized controlled trials over the years [13]. Limited evidence from these trials, each with a small patient cohort, and their meta-analyses indicates that antibiotic prophylaxis is effective to prevent implant failure or the development of postoperative infections with a NNT ranging from 14 to 143 [10–13].

The use of antibiotic prophylaxis to prevent surgical site infections is well established in orthopedic and general surgery, with evidence-based guidelines in place [22]. However, its use in oral surgical procedures, particularly oral implant placement, is less clear. The 4th European Association for Osseointegration (EAO) Consensus Conference 2015 advised against antibiotic prophylaxis for low-risk interventions in healthy patients, suggesting it is beneficial only in complex cases (e.g., patients requiring grafting procedures or immediate placement in extraction sockets) and/or compromised patients, without specifying antibiotics or dosing regimens [14]. In contrast, the Spanish Society of Implants (SEI) recommends prescribing antibiotic prophylaxis even in routine situations for healthy patients, advising a single aminopenicillin dose 1 h before surgery [17]. For immediate dental implants (even without chronic infection of the tooth to be extracted) or sinus lifts, SEI advocates for postoperative continuation of the prophylactic antibiotic regimen for up to 9 days [17]. This approach contrasts with current principles in general surgery and periodontal plastic surgery, which state that there is no evidence to support postoperative antibiotic prophylaxis and thus no indication for it [23]. In Germany, no comprehensive guideline exists for antibiotic prophylaxis in implant surgery. Although routine use is debated, some guidelines recommend prophylaxis for high-risk patients. The S3 guideline advises a single preoperative antibiotic dose for diabetic patients undergoing dental implantation [16]. Similarly, systemic antibiotic prophylaxis is recommended for patients on antiresorptive therapies, such as bisphosphonates or denosumab, during jaw surgeries, including tooth extractions and implant placements [24, 25]. This also applies to patients who have undergone jaw radiotherapy [26].

Given the unclear and sometimes conflicting information in the literature regarding antibiotic prophylaxis in dental implant surgery, our study aimed to characterize antibiotic prescribing habits for oral implant surgery in Germany. Among the participating implantologists, 74.8% administered antibiotic prophylaxis to all their patients, indicating that roughly three-quarters of German implantologists routinely prescribe antibiotic prophylaxis. In comparison, studies from other European countries show that only 23% of implantologists in Turkey and 44% in the Netherlands routinely prescribe antibiotics [19, 27]. In contrast, the figures for the UK and Sweden are similar to those in Germany, at 72% and 74%, respectively [28, 29].

Our results suggest that the minority of implantologists who use antibiotic prophylaxis on a case-by-case basis consider patient-related and procedural risk factors. The strongest predictors of prophylactic antibiotic use were the patient’s diagnosis of cardiovascular disease, whether bone augmentation was performed, and the timing of dental implant insertion—immediate, early, or delayed—with ORs between 3.1 and 7.0. Factors such as the patient’s age had no influence.

Known risk factors for implant failure include smoking, systemic conditions (e.g., uncontrolled diabetes, osteoporosis, immune disorders), medication use (e.g., bisphosphonates), and previous radiation treatment [30–32]. We did not evaluate all these factors to avoid overloading the questionnaire, which might have affected the response rate. Furthermore, we aimed to observe the implantologists’ practices with minimal influence, as asking about specific risk factors might have sensitized them and influenced their use of antibiotic prophylaxis.

German implantologists use a narrow range of antibiotics, primarily aminopenicillins (with or without a beta-lactamase inhibitor) for two-thirds of prescriptions and clindamycin for the remaining third. These drugs were historically recommended for endocarditis prophylaxis, another important indication for prophylactic antibiotic use in dentistry. However, the 2023 European Society of Cardiology guidelines no longer recommend clindamycin for endocarditis prophylaxis due to its association with adverse events, including fatal and non-fatal *Clostridioides difficile* infections. This revised risk–benefit assessment may also be relevant for the perioperative use of clindamycin in implant surgery, especially as clindamycin is less effective in preventing implant failure than other antibiotics [33] and even failed to show preventive efficacy compared to placebo [34, 35]. Additionally, the use of clindamycin during sinus augmentation has been associated with an increased risk of graft failure compared to amoxicillin [36]. Although clindamycin use was reduced in bone augmentation cases compared to routine interventions, its overall frequent use, as observed in our study, is not justified given the current evidence.

Common bacteria causing implant infections include streptococci, anaerobic gram-negative rods, anaerobic gram-positive cocci, and anaerobic gram-negative cocci. Bacterial samples obtained during maxillary sinus elevations predominantly consisted of Streptococcus species (45%), mainly Streptococcus viridans, followed by Staphylococcus species (25%), Enterobacteriaceae (25%), and Haemophilus influenzae (5%) [37]. The most effective antibiotics in that study were ampicillin, amoxicillin–clavulanic acid, and ciprofloxacin [37], supporting the preferred use of aminopenicillins for antibiotic prophylaxis. Determining an alternative antibiotic for prophylaxis in implant surgery if aminopenicillins are contraindicated, such as due to allergies, remains challenging. Based on a study comparing single doses amoxicillin with clarithromycin in terms of surrogate endpoints (specific proinflammatory cytokine and chemokine concentrations in peri-implant crevicular fluid and gingival crevicular fluid) [38], the Spanish Society of Implants recommends the macrolide clarithromycin in its guidelines [17].

Our study revealed that more than three-quarters of patients received postoperative antibiotics despite no clear evidence that this improves outcomes in terms of osseointegration, reduced incidence of postoperative infections, or implant survival. In one study, patients treated with postoperative antibiotics experienced greater peri-implant vertical bone loss up to the 6-month follow-up but showed less bone loss in subsequent intervals up to 60 months [39]. Another study on healthy patients undergoing implant surgeries without additional bone grafting found that systemic postoperative antibiotics did not influence peri-implant crestal bone change or postoperative morbidities compared to preoperative single-dose antibiotics [40]. Additionally, postoperative antibiotics were not superior to a preoperative single-dose regimen regarding postoperative infection rates or implant failure in patients undergoing implant surgery, including those with guided bone regeneration but excluding those requiring sinus lift surgery [41]. The marginal benefits, if any, of postoperative antibiotic prophylaxis are outweighed by the risks of adverse effects on the gastrointestinal flora and the accelerated development of bacterial resistance. The Spanish Society of Implants provides only a low-level grade D recommendation for postoperative antibiotic prophylaxis, limited to special situations such as immediate dental implantations and implantations with sinus lifts [17].

We found that the type of training dentists undergo to become implantologists significantly influences their use of antibiotic prophylaxis. Developing a national or European guideline on antibiotic prophylaxis in implant dentistry, which is currently lacking, would standardize its evidence-based use in Germany.

A limitation of the study is that we did not record known patient-related risk factors for implant failure, such as diabetes mellitus, bone antiresorptive therapies, immunosuppression, or smoking. We excluded these factors for several reasons: (a) they have been extensively studied, (b) simplifying the survey was intended to encourage participation from implantologists and improve response rates, and (c) we aimed to minimize the survey’s impact on the prospective observation of prescription practices. However, unlike most previous surveys on the topic, our method of prospectively collecting data from at least 10 consecutive cases from each implantologist enabled us to gather and analyze real-world data.

## Conclusions

Evidence suggests that antibiotic prophylaxis in oral implant surgery helps prevent periimplantitis and implant loss. However, clinical practice guidelines are largely missing, leaving several questions unanswered for German implantologists: which patient groups, based on patient and procedural risk factors, should receive antibiotic prophylaxis? Which antibiotics are most effective? What dosing scheme is appropriate? Comparing the use of antibiotic prophylaxis in German implantology practice with clinical evidence leads to the following conclusions:

- The widespread use of clindamycin, as observed in our study, is unjustified. Clindamycin carries a significantly higher risk of osseointegration failure and infection—up to six times higher—compared to aminopenicillins [17]. Clindamycin also has a higher rate of adverse reactions: 13 fatal and 149 nonfatal reactions per million prescriptions, versus 0 fatal and 23 nonfatal reactions per million prescriptions for amoxicillin [42]. For patients contraindicated for aminopenicillins, macrolides such as azithromycin or clarithromycin are recommended, similar to recent endocarditis prophylaxis guidelines [17, 43]. However, their efficacy in implantology has not yet been proven in RCTs.
- Postoperative antibiotic prophylaxis, commonly practiced, is unnecessary according to evidence [44]. A single preoperative dose is equally effective in preventing periimplantitis and improving implant retention, without the additional risk of antibiotic-associated collateral damage to the patient’s microbiota and resistance development [44].
- Comprehensive guidelines should be established to ensure consistent, evidence-based use of antibiotic prophylaxis. This would reduce the variability in the use of antibiotic prophylaxis associated with the specific training that dentists receive as implantologists, as highlighted in our study.

## Data Availability

No datasets were generated or analysed during the current study.

## Abbreviations

BLI: Beta-lactamase inhibitor
CI: Confidence interval
DGOI: German Society for Oral Implantology
EAO: European Association for Osseointegration
IQR: Interquartile range
NNT: Number needed to treat
OR: Odds ratio
RCT: Randomized clinical trial
SD: Standard deviation
SEI: Spanish Society of Implants
STROBE: Strengthening the Reporting of Observational Studies in Epidemiology
